# Host protective roles of type 2 immunity: Parasite killing and tissue repair, flip sides of the same coin

**DOI:** 10.1016/j.smim.2014.06.003

**Published:** 2014-08

**Authors:** Judith E. Allen, Tara E. Sutherland

**Affiliations:** Centre for Immunity, Infection & Evolution, School of Biological Sciences, University of Edinburgh, Edinburgh, UK

**Keywords:** IL-4Rα, Interleukin-4 receptor alpha, ILC, innate lymphoid cell, TSLP, thymic stromal lymphopoietin, AAMφ, alternatively activated macrophage, Helminths, Th2 cells, Wound repair, Macrophages, Eosinophils, IL-4Rα

## Abstract

Metazoan parasites typically induce a type 2 immune response, characterized by T helper 2 (Th2) cells that produce the cytokines IL-4, IL-5 and IL-13 among others. The type 2 response is host protective, reducing the number of parasites either through direct killing in the tissues, or expulsion from the intestine. Type 2 immunity also protects the host against damage mediated by these large extracellular parasites as they migrate through the body. At the center of both the innate and adaptive type 2 immune response, is the IL-4Rα that mediates many of the key effector functions. Here we highlight the striking overlap between the molecules, cells and pathways that mediate both parasite control and tissue repair. We have proposed that adaptive Th2 immunity evolved out of our innate repair pathways to mediate both accelerated repair and parasite control in the face of continual assault from multicellular pathogens. Type 2 cytokines are involved in many aspects of mammalian physiology independent of helminth infection. Therefore understanding the evolutionary relationship between helminth killing and tissue repair should provide new insight into immune mechanisms of tissue protection in the face of physical injury.

## Highlights

• Type 2 immunity is associated with both helminth infection and responses to injury.
• Pathways involved in tissue repair and helminth immunity overlap.
• The IL-4Rα is central to accelerating both repair and helminth control.
• Adaptive immunity contributes to more rapid wound repair.

## 1 Introduction

Multicellular metazoan parasites of mammals, also known as helminths, typically induce a Th2-type (type 2) immune response in the infected host. Type 2 immunity is a highly complex multi-cellular, multifactorial system characterized by the cytokines IL-4, 5, 9, 10 and 13 [1]. These cytokines are produced by Th2 lymphocytes, but also by a range of innate immune cells including basophils, eosinophils, mast cells and innate lymphoid cells (ILCs). The IL-4R alpha chain (IL-4Rα), a component of both the IL-4 and IL-13 receptor, is fundamental to type 2 immune function [1], with expression and engagement of IL-4Rα on immune effector cells (e.g. macrophages, B cells) and tissue cells (e.g. smooth muscle, epithelial cells) dictating the outcome of a type 2 immune response. Details of type 2 immunity are constantly emerging, including the discovery of new cells, such as ILCs, which are central to type 2 immune function. These processes, particularly as they apply to helminth immunity have been reviewed recently [1], [2], [3].

The triggers that initiate a type 2 immune response have been under intense investigation for many years with little consensus on the essential pathways that lead to a robust response. However, recently there has been an emerging literature on the critical role of mucosal barriers. In particular, epithelial cells release alarmins, IL-25, IL-33 and thymic stromal lymphopoietin (TSLP) [4], all three of which promote Th2 immunity. It is becoming increasingly apparent that we have a robust cytokine alert system to tissue injury that will activate a type 2 immune response in the absence of a more dominant type 1 trigger [5]. Recently, Patel et al. [6] directly linked cellular damage to the induction of protective immunity against a gastrointestinal nematode by demonstrating that release of extracellular adenosine was responsible for inducing the Th2 response. Consistent with this, mast cells, which act as sentinels of injury [7], and other innate cells such as eosinophils and basophils, can readily produce IL-4 which has long been implicated in the generation of a type 2 immune response [8].

Tissue injury alone does not always generate a type 2 response. Additionally, helminth products themselves can induce Th2 responses in the absence of injury [9], [10]. Thus, over evolutionary time it seems that mammals have learned to recognize the presence of potentially damaging large multicellular organisms, and initiate a response that largely resembles a reaction to tissue injury. This association between tissue injury and infection with metazoan parasites makes sense. Metazoan parasites do not typically replicate in their hosts; instead they enter as larval stages, frequently migrate through tissues to their established niche, grow through developmental stages to sexual maturity, mate and release offspring to infect a new host. Faced with these large tissue migrating invaders, a pro-inflammatory type 1 response, although potentially damaging to the parasite, is even more likely to damage the host. Thus, a response that limited type 1 inflammation and facilitated wound healing would be beneficial. Ideally, this same response, where possible, would contribute to parasite control, either by limiting their numbers, hindering their development, restricting their motility, or preventing new incoming infections. Indeed, this does appear to be the case. The overlap between injury repair pathways and parasite control is striking, as this review will illustrate. The relationship between parasite killing and wound repair is also reflected in the association of fibrosis with helminth infection [11]. The requirement for rapid tissue repair following parasite migration, can necessitate aggressive matrix deposition, the natural consequence of which is formation of scar tissue and fibrosis [12]. IL-13 appears to be the critical type 2 cytokine involved in the fibrotic response, both through direct effects on collagen production and deposition and indirect effects in promoting TGFβ-mediated repair [13]. IL-13 and/or IL-4, which both use the IL-4Rα chain, are also central to resistance against many if not most helminth infections [1].

**Fig. 1:**
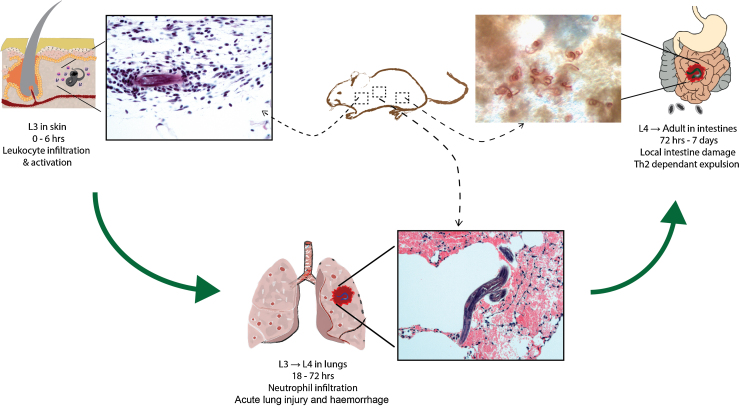
Life cycle of *N. brasiliensis* in mice, demonstrating sites where tissue injury occurs. Stage 3 larvae (L3) infect the host by penetrating the skin resulting in local infiltration of host neutrophils and esoinophils. L3s enter blood vessels (∼6 h post-infection) and migrate to the lung bursting through capillaries (∼18–72 h) where, in the parenchyma, L3s mature to L4s. Damage caused by larval migration and neutrophilic inflammation leads to hemorrhage and acute lung injury. After approximately 48 h, larvae break through into the airways, are coughed up, swallowed and enter into the intestine where parasites mature and produce eggs (72 h). Adults that reside in the intestine cause local tissue damage and inflammation before being expelled in a highly Th2-dependent manner.

Infection with the rodent gastrointestinal nematode, *Nippostrongylus brasiliensis* has proved a powerful and useful model to evaluate both control of nematode numbers and repair of damage caused by nematode migration. Throughout this review, *N. brasiliensis* will be used to illustrate the dual function of so many core components of the type 2 immune response, although other models will be described where relevant. As with the related hookworm parasites of man, *N. brasiliensis* larvae invade by penetrating the skin and entering the blood vessels where they are swept to the lung (Fig. 1). Parasites burst from the capillary bed into the lung parenchyma, causing substantial bleeding. Once in the lung, the larvae undergo one molt and within 48 h move into the airways and trachea, where they are coughed up and swallowed by the host. In the gastrointestinal tract parasites reach sexual maturity and produce eggs. Atypical of many helminth infections, *N. brasiliensis* in mice is a relatively acute infection and depending on parasite/host strains, adult worms are expelled from the gut in 1 to 2 weeks. Expulsion is highly Th2 dependent, with a critical role for Stat-6 and the IL-4Rα [14], responses that are also needed for protection from re-infection [15]. Whilst larval migration through the lung causes considerable damage, the tissue is rapidly repaired in a process dependent on type 2 activated macrophages [16]. Nonetheless, the progressive airway remodeling that occurs can lead to deficits in lung function and after some 50 days post infection, the lung in all strains of infected mice exhibit an emphysematous morphology of unknown origin [17], [18].

## 2 Alerting the immune system to injury

**Fig. 2:**
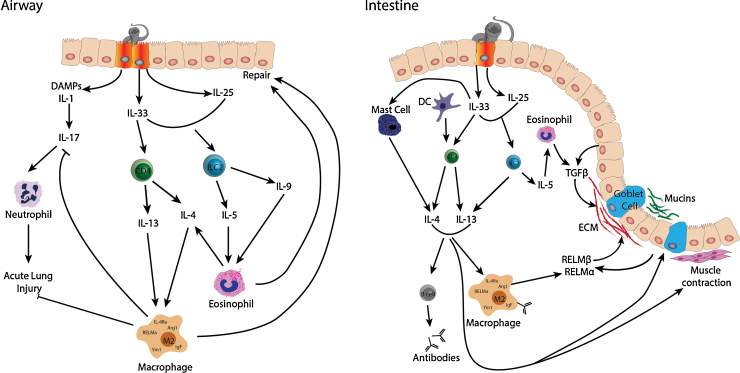
Effector molecules involved in type 2 immune responses and host repair following *N. brasiliensis* infection. While the pathways involved in immune-mediated clearance and repair of tissue damage can be applied to infection of most helminths, the effector molecules depicted here apply specifically to infection with *N. brasiliensis*. As larvae pass through the lung, acute lung injury ensues following danger associated molecular patterns (DAMPs) and IL-1 secretion from airway epithelial cells, which in turn triggers IL-17 production and recruitment of neutrophils. Neutrophil influx in combination with mechanical damage from migrating larvae leads to injury and tissue hemorrhage. Concurrently, alarmins IL-33 and IL-25 are secreted from epithelial cells leading to recruitment and activation of both CD4+ T cells and innate lymphoid cells. Type 2 cytokines IL-4, 13, 9 and 5 increase in the tissue leading to eosinophilia, which in turn contributes to the pool of IL-4. Macrophages in the lung become alternatively activated by engagement of the IL-4Ra and secret factors such as RELMα, Ym1, arginase (Arg1) and insulin growth factor (IgF) that inhibit IL-17 production limiting further tissue damage. Additionally M2 macrophages simultaneously facilitate repair of the lung, along with eosinophils. Responses that occur in intestine follow a similar trend, although the damage from infection is not only initiated by parasite migration but also feeding on the intestine wall. Alarmins together with dendritic cells and mast cells stimulate a type 2 immune response resulting in alternative macrophage activation. AAMφ not only secrete mediators, like RELMα that help toward repair of the tissue and extracellular matrix remodeling, but are also involved in parasite killing following activation by parasite-specific antibodies secreted by B cells. Critically, epithelial cells and goblet cells play a key role in repair of tissue by generating factors like TGFβ, RELMα and RELMβ which contribute to extracellular matrix turnover. Approximately 1 week post infection with *N. brasiliensis*, the adult parasites are cleared from the intestine in a type 2 dependent manner involving IL-13-driven muscle contraction and increased mucus production in the epithelium. It is important to note that while TSLP effector function is redundant for host-protection against *N. brasiliensis* infection it is an important factor in other helminth models as discussed in Section 2.3.

IL-33, IL-25 and TSLP alert the immune system to injury and promote the development of a type 2 immune response. Each of these molecules illustrates the intimate relationship between parasite control and injury repair (Fig. 2).

### 2.1 Interleukin-33

IL-33 is a member of the IL-1 family and its receptor, ST2, is expressed on mast cells, Th2 cells [19], ILC2s [20], [21] and can be highly upregulated on macrophages by Th2 cytokines [22]. In keeping with its designation as an alarmin, IL-33 is released in a bioactive form by dying cells [23] and a key mechanism by which mast cells respond to injury is via recognition of IL-33 [7]. IL-33 promotes multiple aspects type 2 immunity [19] and this has been documented in the context of helminth exposure through intravenous administration of *Schistosoma mansoni* eggs, one of the most potent inducers of type 2 immunity known. Mice that lack ST2 fail to develop primary Th2 responses or form Th2-dependent lung granulomas around the eggs [24]. Thus, the evidence that IL-33 acts by alerting the immune system to injury and induces type 2 immune responses is strong. The response elicited by IL-33 also impacts on the repair process and this is documented by accelerated repair of incisional wounds following IL-33 administration [25] and emerging evidence for IL-33 in epithelial restoration and mucosal healing in the gut [26]. The promotion of type 2 cytokines and healing, also means IL-33 contributes to fibrosis in a variety of experimental models [27], [28].

As a potent initiator of Th2 responses, it was logical to test the role of IL-33 in *Trichuris muris*, a nematode infection that is strictly dependent on Th2 immunity for parasite expulsion from the intestine. Humphreys et al. [29] demonstrated that IL-33 mRNA was elevated early following infection, and that administration of recombinant IL-33 was sufficient to accelerate clearance of the parasite. The importance of IL-33 for parasite control was also demonstrated for *N. brasiliensis*, where IL-33 is needed in both primary and secondary infection to promote expulsion [30]. The effect on worm expulsion was due to the ability of IL-33 to promote IL-13 production by both ILCs and CD4+ T cells, which in turn increases production of the anti-worm effector molecule RELMβ by intestinal epithelial cells. In this same study, IL-33 deficiency led to greater hemorrhaging at day 3 post infection, along with reduced eosinophil recruitment to the lung. Thus IL-33 is critical for worm expulsion, while also minimizing host damage early in infection [30].

### 2.2 Interleukin 25

IL-25 is a member of the IL-17 cytokine family produced by epithelial cells, amongst other cell types, and is likely a sensor of epithelial disruption [4]. Like IL-33, IL-25 induces the production of type 2 cytokines by ILCs. As a direct result, type 2 cytokine responses and parasite expulsion are delayed in *N. brasiliensis* infected IL-25 deficient mice [31]. Further, delivery of recombinant IL-25 into RAG-deficient mice is sufficient to mediate parasite expulsion [31], [32]. Similarly, when mice normally susceptible to *T. muris* infection were treated with IL-25, they were able to effectively expel the parasite, while IL-25 deficiency on the genetically resistant background prevented worm expulsion [33]. Similar methods revealed that IL-25 protected against infection with *Trichinella spiralis*, including reducing both the worm burden in the intestine and the number of larvae in the muscles [34]. Together these studies demonstrated the potency of IL-25 as an anti-nematode effector. Importantly, in the *T. muris* study, Owyang et al. demonstrated that IL-25 was able to limit the intestinal inflammation and tissue damage in the colon associated with this infection demonstrating the dual roles of this alarmin cytokine [33]. Independently of helminth infection, the anti-inflammatory properties of IL-25 that protect against gut damage are also reflected in studies of type 1 induced colitis [35], [36]. Not surprisingly, because of its ability to enhance type 2 responses, IL-25 also promotes allergic responses that themselves can lead to tissue damage and remodeling [37] as well as fibrosis [38], the consequence of aggressive wound repair.

### 2.3 Thymic stromal lymphopoietin (TSLP)

TSLP is a member of the IL-2 cytokine family expressed predominantly by epithelial cells. Expression of TSLP is constitutive in the lung and gut where it is believed to suppress inflammatory type 1 responses and promote type 2 responses [39]. TSLP can be further induced upon tissue injury and is associated with protection of mucosal surfaces [39]. However, its expression is not limited to the barrier surfaces, but TSLP is also induced in response to damage or injury in other tissues such as the liver [40] and the central nervous system [41]. The ability of TSLP to promote Th2 responses is in part due TSLP receptor expression on dendritic cells [42]. Beyond the promotion of Th2 cells, TSLP amplifies type 2 effector responses, for example by enhancing the polarizing effects of IL-13 on macrophages [43].

An important role for TSLP in resistance to helminth infection was shown by infection of mice with *T. muris*, in which TSLP blockade made resistant mice susceptible and TSLP receptor deficient mice have enhanced worm burdens [44]. It was somewhat surprising, therefore, when other models of helminth infection including *N. brasiliensis* failed to demonstrate a role for TSLP in host protection [45]. Massacand et al. [45] were able to explain this discrepancy by demonstrating that the major function of TSLP during *T. muris* infection was to suppress IL-12p40. Because *N. brasiliensis* and *H. polygyrus* parasites directly inhibit host IL-12p40, a feature typical of many nematode infections, the contribution of TSLP was redundant. Thus a major mechanism by which TSLP promotes type 2 immunity is likely to be through the suppression of type 1 responses. Nonetheless, in the appropriate context, this allows the host to mount an effective anti-helminth response.

Evidence for a role of TSLP in wound repair comes from studies demonstrating reduced fibrosis in the absence of TSLP or its receptor. In liver fibrosis induced by deposition of *S. mansoni* eggs, fibrosis was attenuated in TSLP receptor deficient mice and this was associated with decreased IL-13 production [46]. Although fibrosis is a negative consequence of poorly regulated repair, the functions of TSLP that have been elucidated in fibrosis models provide direct evidence for its repair function. For example, in a model of atopic dermatitis, TSLP directly promotes fibrocytes, cells that circulate in the peripheral blood and produce connective tissue proteins, to produce collagen. Neutralization of TSLP or genetic deletion of TSLPR resulted in a significant reduction in the number of fibrocytes and in skin fibrosis. Interestingly, this tissue remodeling function of TSLP was independent of IL-13 and likely due to TSLP receptor expression on fibrocytes [47].

### 2.4 Innate lymphoid cells

All three alarmins described above promote Th2 responses in a large part through their ability to induce IL-5 and IL-13 production from ILCs [48]. ILCs are distinct groups of innate cells that display transcriptional and functional attributes directly analogous to the adaptive T helper effector cells, Th1, Th2, Th17. Specifically ILC2s, like Th2 cells, can produce IL-5, IL-9 and IL-13 and require GATA-3 for lineage commitment [reviewed in 49]. Furthermore, ILC2s are an essential component for the induction of adaptive Th2 cells after lung allergen exposure, as was shown recently by the ability of ILC derived IL-13 to promote migration of CD40+ DCs to the draining lymph nodes [50]. Early discovery of ILC2s was in the context of *N. brasiliensis* infection [31], [51] and ILC2s were subsequently shown to promote expulsion of the parasite from the gastrointestinal (GI) tract [20], [21], [32]. In this infection context, IL-13 producing ILC2s promotes goblet cell mucus secretion and smooth muscle contraction, processes that mediate the expulsion of helminth parasites.

The *N. brasiliensis* model has recently been used to elegantly illustrate the contribution of ILCs to both lung repair and worm expulsion, while simultaneously revealing a critical role for IL-9 in both these processes [52]. Turner et al. demonstrate that during infection, ILC2s are the dominant IL-9 producing cell in the lung. In mice lacking the IL-9 receptor, the lungs fail to repair appropriately following larval migration, with prolonged micro-bleeding and the subsequent emphysema-like damage dramatically increased. In addition, worm expulsion from the GI tract is delayed in the absence of the IL-9 receptor on hematopoietic cells. Therefore, the authors conclude that ILC-derived IL-9 promotes lung repair and worm expulsion. In the absence of IL-9 there is significantly reduced eosinophils and IL-4Rα-activated macrophages as measured by RELMα, presumably because a reduction in ILCs would lead to insufficient IL-5 and IL-13 production and a failure to induce a sufficient adaptive Th2 response. ILCs in this setting also produce amphiregulin that might contribute to repair (discussed below).

## 3 Macrophages

Many of the tissue protective functions of IL-4 or IL-13 produced by ILCs, Th2 cells or other innate cells are likely to be carried out by macrophages. Macrophages express receptors for both IL-4 and IL-13 and their receptors share the common IL-4Rα chain, which is central to most type 2 effector responses [1]. For the purposes of this review, alternatively activated macrophages (AAMφ) are specifically defined as cells that respond to signaling through the IL-4Rα, following the original definition by Siamon Gordon [53].

### 3.1 Macrophages and helminth killing

There is a new emerging literature on the ability of macrophages to kill nematodes in a Th2 context [54], [55], [56]. Despite evidence that the granulomas around dying nematodes are predominantly macrophages [57] and that macrophages can kill in vitro [reviewed in 58], it has taken surprisingly long for this data to emerge in vivo. This may be due to the difficulties in effectively depleting macrophages or because of redundant mechanisms and the fact that macrophages apparently do not act alone. For example, Bonne-Anne et al. recently demonstrated that both human and mouse macrophages collaborate with neutrophils to kill larvae of the nematode *Strongyloides stercoralis* [59]. In their studies, complement, neutrophils and macrophages are all involved. Although either neutrophils or macrophages need to be in contact with the worm, the cell types can be separated from each other. So soluble communication between the cells is needed but either one can kill. While in vivo studies showed that naïve macrophages could kill parasites given enough time, AAMφ are the most effective. Therefore, IL-4Rα activation of macrophages accelerates the parasite killing process.

A seminal study by Anthony et al. demonstrated that clodronate-mediated monocyte depletion prevented worm expulsion during secondary *H. polygyrus* infection [60] and more recently, antibody has been shown to play a critical role in activating macrophages to kill in *H. polygyrus* infection [55]. Critically, the effects of macrophages may not always be direct. For example in *N. brasiliensis* infection, macrophage depletion alters intestinal smooth muscle function that is involved in worm expulsion [61]. All three of these studies suggest arginase as an important anti-parasite mediator (discussed below).

Although depletion studies have strongly implicated macrophages in killing or expulsion, the specific contribution of the IL-4Rα has been less clear. Results with LysMCre deletion of the IL-4Rα have been inconsistent, perhaps due to incomplete depletion in these mice and the propensity for the IL-4Rα positive cells to outcompete the gene-deleted cells in high IL-4 environments [62]. Furthermore, with the exception of arginase, the evidence that IL-4 induced proteins contribute to worm attrition is still lacking. Indeed, RELMα, one of the most highly induced proteins in AAMφ, may inhibit worm killing due to it's ability to negatively regulate type 2 immunity [63], [64].

There is no doubt that the mechanisms by which AAMφ contribute to worm control will be highly varied, from direct targeting of the worm, to the regulation of glucose transport in the intestine that alters epithelial cell function [65], and to recruitment of eosinophils [22], [66]. The broad phylogenetic diversity of helminth parasites, their distinct host niches, migratory routes, and mechanisms of host manipulation make finding a common answer to “How do macrophages kill worms?” very unlikely. Similarly, no single pathway will be exploited by AAMφ to repair tissue. The interesting common ground may be the intersection between worm killing and tissue repair, where the same pathways, e.g. epithelial cell turnover or eosinophil recruitment, lead to different but not mutually exclusive outcomes.

### 3.2 Alternatively activated macrophages in repair

There is a large and growing literature documenting that a reprogramming of macrophages away from an M1 phenotype promotes tissue repair and regeneration [reviewed in 67]. These ‘repair’ macrophages are typically called M2, but this encompasses an enormous range of potential phenotypes, and the specific contribution of IL-4Rα signaling to repair and regeneration processes still needs to be elucidated. The evidence that IL-4Rα signaling in macrophages contributes to repair is mainly circumstantial although increasingly strong. The IL-4Rα dependent production of arginase is one of the earliest and best examples, and the contribution to tissue remodeling and repair is a well established property of arginase 1, which is discussed below. These properties also explain the frequent association of arginase with fibrosis and in particular asthma, where it is believed to contribute to pathological tissue remodeling [68].

In addition to arginase, Ym1/2 (*chi3l3/chi3l4*) and RELMα (*retlna*) are highly but transiently unregulated in response to incisional wounding in an IL-4Rα dependent manner [69]. Although no specific repair function has been defined for the chitinase-like proteins including Ym1, they bind extracellular matrix [70] and are frequently identified in injury settings [69], [70], [71]. There is considerably more evidence for a pro-repair role for RELMα, which has documented angiogenic properties [72]. However, the contribution of RELMα to be repair may be highly complicated by it's ability to suppress Th2 responses, so although it apparently has direct repair functions, it also acts in a negative feedback loop to control fibrosis [63], [64].

Many other repair proteins are regulated by IL-4 or IL-13 in macrophages contributing to the evidence that tissue protection is a key function for AAMφ. For example, transcriptionally we observed that extracellular matrix degrading matrix metallo-proteases (MMP) are actively down regulated by the IL-4Rα in macrophages, while their inhibitors (TIMP1 and TIMP2) are upregulated [22]. Similarly, we observe IL-4Rα induction of insulin like growth factor (IGF-1) during helminth infection [22] as previously demonstrated in vitro [73]. IGF-1 has a long established role in repair in part through its ability to stimulate the proliferation and survival of fibroblasts and myofibroblasts and promote matrix production. The importance of both IGF-1 and AAMφ in tissue repair was illustrated by Chen et al. [16]. In that study, IGF-1 producing AAMφ were needed to repair the damage caused by lung migrating *N. brasiliensis* larvae.

A recent study demonstrated that AAMΦ take up collagen aggressively through the mannose receptor and degrade it, potentially identifying a specific function for AAMΦ in repair [74]. Although more direct evidence is still needed, there is little doubt that macrophages activated via the IL-4Rα contribute to repair. The future challenge will be to identify the specific functions of AAMφ-derived repair molecules in specific settings and functions. This will require testing the quality and rate of repair in macrophage specific deletions including the IL-4Rα itself.

### 3.3 Alternatively activated macrophages in regulating inflammation

Although the specific roles Th2 induced proteins play in the complex orchestra of tissue repair and remodeling is still being established, an important contribution may be to rapidly shut down the early inflammatory response to injury in order to allow wound repair to progress [75]. Thus, the well-documented anti-inflammatory nature of AAMΦ is one important feature that is likely central to their wound repair functions. This makes evolutionary sense in the context of immunity to helminths: a host infected with macroparasites would want to repair any damage caused by the pathogen but also avoid the damaging consequences of mounting an inflammatory response to a large tissue migrating parasite.

The data supporting an anti-inflammatory role for AAMφ has been largely based on the evidence that AAMφ are important sources of down-regulatory cytokines including TGF-β [76], [77], PGE2 [78] and the IL-1 receptor antagonist [77], [79]. The chemokine expression profile is also strongly associated with a non-inflammatory role [80] and with specific down-regulation of key pro-inflammatory chemokines by IL-4 [22], [79], [81]. Strong evidence that AAMΦ have a combined anti-inflammatory/wound healing function comes from a study of *S. mansoni* infection in mice that lack the IL-4Rα specifically on macrophages and neutrophils and thus completely lack AAMΦ but have otherwise intact Th2 responses [82]. Following *S. mansoni* infection, these mice die from overwhelming inflammatory responses in the intestine and leakage of bacteria into the blood. The data suggests that in the absence of AAMΦ, these mice were unable to repair the damage caused by egg migration through the intestinal wall.

TGF-β nicely illustrates that a single protein can be both anti-inflammatory and a critical mediator of repair, and its damaging sequela, fibrosis [83]. Similarly, the AAMΦ product 12/15 lipoxygenase is needed for effective wound repair [84] but attenuates pro-inflammatory macrophage activation [85]. Although IL-10 is very strongly associated with an M2 phenotype, and is often listed as a prototypic cytokine associated with alternative activation [54], [77], it is not specifically an IL-4Rα dependent macrophage product. RNAseq analysis comparing WT to IL-4Rα−/− macrophages had sufficient depth of coverage to be able to say categorically that IL-10 is not produced by F4/80 macrophages in the context of filarial nematode infection [22]. Similarly, the important source of IL-10 following both hookworm migration through the lung and filarial nematode infection appears to be T cells and not AAMΦ [16], [86]. This is consistent with very recent data in which the critical anti-inflammatory roles of macrophages in the gut are mediated by their ability to respond to IL-10 rather than the production of IL-10 [87]. IL-10 production by macrophages may be more related to the ability of classical pro-inflammatory macrophages to self-regulate [88].

### 3.4 Tissue resident vs. recruited macrophages

The recent paradigm shift in our understanding of macrophage biology [89] adds a new layer of complexity to the contribution of AAMΦ to infection control and repair. Until very recently, it was generally understood that most tissue macrophages are derived from the bone marrow via blood circulating monocytes. We now realize that in fact many tissue-resident macrophages including the spleen, serous cavities and liver are established in the tissue pre-natally by embryonic precursors that are sustained by a process of continual self-renewal [reviewed in 89]. Alveolar macrophages are similarly maintained throughout life by proliferative self-renewal but are first established shortly after birth by a fetal monocyte population [90]. Dermal and intestinal macrophages are exceptions as they are of bone marrow origins [91], [92], [93].

We recently made the unexpected discovery that IL-4 can induce macrophages to proliferate well beyond levels required for steady state renewal. We were investigating the contribution of blood monocytes to helminth killing in a model of filarial nematode infection in which the adult parasites reside in the pleural space. To our surprise, monocyte depletion had no impact on the very large increase in macrophage numbers at the site of infection [94]. We were able to establish that in this model IL-4 induces a novel form of ‘inflammation’ in which the increase in cell number is due to proliferation rather than blood cell recruitment. We observed similar proliferative expansion in the peritoneal cavity of mice infected with the GI nematode, *H. polygyrus* [62]. We were also able to establish that when macrophages were recruited from the blood, IL-4 was still capable of inducing further proliferation and alternative activation as measured by RELMα, Ym1 and arginase production [94]. It now becomes necessary to establish in each model the relative contribution of resident vs. recruited macrophage populations. Two recent studies in models of schistosomiasis have demonstrated that despite proliferation of resident macrophages during infection, the dominant route for increased macrophage numbers is blood monocyte recruitment not local proliferation [95], [96].

Both recruited and resident macrophages can become alternatively activated by IL-4 [94]. This raises critical questions about the differential function of resident vs. recruited macrophages. Beyond their location, the longevity of many tissue resident macrophages would support a role for maintenance of tissue integrity. Micro-array analysis that compared IL-4-activated macrophages from resident or monocyte-derived origin illustrated that their functions are likely to be highly distinct, with tissue-resident cells taking on a prominent role in maintenance of homeostasis, rather then immune regulation [97]. Although tissue resident cells are likely to have major roles in tissue protection, these functions might be taken over by recruited cells that get activated by type 2 cytokines. We find that IL-4 strongly induces a non-inflammatory phenotype, with the down regulation of chemokine receptors that may be involved in the trafficking of macrophages beyond the infection or injury site [22]. IL-4Rα signaling also actively shuts down pro-inflammatory chemokine production and promotes an eicosanoid environment dominated by anti-inflammatory lipid mediators [22]. The ability of IL-4 to drive macrophage proliferation is evidence of the fundamental non-inflammatory nature of the macrophage response in the context of type 2 immunity. Local proliferation allows expansion of a large effector cell pool, avoiding the need for recruitment of potentially damaging neutrophils and monocytes. However, a proliferative response is necessarily much slower than recruitment from the blood and may be the dominant mechanism only when the risk of microbial insult is sufficiently low (Box 1).

Box 1 **Unanswered Questions**

• What is specific contribution of IL-4Rα signaling to repair and regeneration processes?
• What are the specific properties of IL-4Rα induced products such as RELMa, arginase and YM1 that mediate repair and/or worm killing?
• How do single molecules perform both repair functions and parasite killing? Does the cellular source matter?
• What are the distinct functions of different cell types such as macrophages and epithelial cells, when activated via the IL-4Rα?
• Are there distinct functions for resident vs. monocyte-derived macrophages in repair or helminth control?
• What is specific contribution of eosinophils to repair and regeneration processes?
• What are the circumstances in which adaptive immunity contributes to tissue repair?

## 4 Eosinophils

Through release of toxic granular proteins, eosinophils have classically been viewed as key effector cells in host-defense against helminth parasites but also in pathologies of allergic diseases. While circumstantial data has long associated eosinophils with tissue injury and repair, few studies have explored the relationship directly.

### 4.1 Eosinophils as anti-parasite effector cells

Eosinophils accumulate following nematode infection, largely in response to IL-5, a cytokine not only critical for recruitment but also eosinophil differentiation from the bone marrow [98]. Early studies of IL-5, and by association eosinophils, contributed to the notion that eosinophils form an integral part of the anti-parasite effector mechanism. However, as the years went on, it became clear that while eosinophils could mediate parasite killing, these observations were parasite, stage and tissue specific. In vitro data still provides some of the most compelling evidence that eosinophils participate in helminth destruction and such approaches provided a direct means of showing that eosinophils can attach to the cuticular surface of larvae [99], release damaging mediators [100] and kill worms in antibody and complement dependent fashion [99], [101], [102]. However, demonstrating a role of eosinophil-mediated parasite killing in vivo has been fraught with contradictions in the literature. Discrepancies typically reflect the varying methods of depleting eosinophils or triggering eosinophilia, the type of parasite and stage studied.

In the case of *N. brasiliensis* infection, data demonstrating a role for eosinophil-mediated killing in vivo was relatively straightforward. Mice with high eosinophilia driven by IL-5-overexpression exhibited lower worm burdens following primary infection [103], with eosinophils and/or IL-5 likely mediating their action on migrating larval stages in the skin and lung [104], [105]. Similarly several groups have demonstrated the importance of IL-5 and/or eosinophils in protection against larval stages of filarial nematodes [106], [107], [108]. An intriguing observation is that filarial larvae accelerate their development and reproduce earlier in the face of an eosinophil threat [109]. In contrast to these studies, ablation of IL-5 either through genetic manipulation or antibody treatment has shown little effect on parasite burdens in *Schistosoma mansoni* [110], *Trichinella spiralis* [111] or *Trichuris muris* [112] infection. In some cases reduced eosinophil numbers even enhanced infectivity of Strongylida species [113], [114] consistent with the regulatory properties associated with eosinophils [98].

In addition to IL-5 manipulation, mice genetically deficient in eosinophil chemotactic receptor CCR3 or factor eotaxin-1, or even eosinophil deficient mice (ΔdblGATA or PHIL) have provided additional direct evidence for the importance of eosinophils in generating protective immunity against filarial nematodes [115], [116], [117]. Furthermore, eosinophil peroxidase (EPO) and major basic protein (MBP) are important in controlling infection to some parasites [118] but not others [116] showing a direct role for proteins produced by eosinophils following degranulation.

Aside from the ability to release cytotoxic mediators in the presence of an invading parasite, eosinophils can also regulate important immune mechanisms, thereby adding to host-protective effects against helminths. Eosinophils are a rapid source of IL-4 and IL-13 cytokines during helminth infection. Combined with the ability to process and present antigens [119] it would be easy to speculate that eosinophils contribute to host-parasite immunity through initiation of type 2 responses. Indeed mice deficient in eosinophils generally exhibit reduced type 2 responses [120], [121], [122], [123]. However, in a recent study by Voehringer et al. [124] eosinophil-derived IL-4 was redundant for primary protection against *N. brasiliensis* infection. The specific contribution of eosinophil-derived IL-4 to type 2 immunity still needs to be dissected and may be complicated by differences in mouse genetic backgrounds and model systems used.

Overall these observations leave no doubt that in many settings eosinophils can mediate protective immunity against helminth infection and promote type 2 immunity. However, in light of varied observations using different approaches to deplete eosinophils or eosinophil proteins in vivo, questions still remain as to whether other mechanisms of host-protection play a more dominant role. In the case of *T. spiralis* infection, parasite killing occurred at a greater rate in the absence of eosinophils following a switch in the immune response toward enhanced iNOS production [125], [126]. Such studies highlight the importance of having a detailed understanding of immune responses during infection and not just the absence/presence of one cell type.

### 4.2 Eosinophils during injury and repair

Like type 2 responses and macrophages, eosinophils have also been implicated during repair of damage tissue. However, most information regarding the role of eosinophils in tissue repair has been inferred from studying eosinophils during pathogenic tissue remodeling associated with fibrosis and asthma. Considering eosinophils were reported in 1977 to localize to sterile tissue sites [127], it is surprising that there have been so few studies showing a direct role for eosinophils in tissue repair. In fact, some of the most compelling evidence has come only in the last 2 years in two studies which describe a clear mechanism through which eosinophils can promote regeneration of damaged tissue [128], [129]. The liver has an extraordinary ability to regenerate after injury and partial hepatectomy, through well-orchestrated timely events that lead up to proliferation of heptocytes. Little is known about the immune responses that lead up to the proliferative burst of hepatocytes. In a study by Goh et al. [128], mice deficient in eosinophils (ΔdblGATA) exhibited a 50% reduction in hepatocyte proliferation following CCl_4_-induced liver damage. In this model, eosinophil-derived IL-4 was critical in generating IL-4Rα-dependent proliferation of hepatocytes, directly linking eosinophil-initiated type 2 responses to liver regeneration. Similar responses could be attributed to muscle regeneration, whereby rapid eosinophil recruitment follows injury and IL-4 secreted by eosinophils activates muscle resident fibro/adipocyte progenitors [129]. Together these studies illustrate critical roles for eosinophils and type 2 cytokines in tissue regeneration.

Helminth infection and host damage go hand in hand, and one of the first responders to the ensuing type 2 response is eosinophils. It would be reasonable to postulate from studies in tissue regeneration, that eosinophils during helminth infection have a role to play in host repair. Eosinophils are a rapid source of type 2 cytokines during helminth infection, and while eosinophil-derived IL-4 may not be essential for parasite killing [124], it may well be involved in repair following helminth migration. Consistent with this hypothesis, failure to repair the lungs of IL-9R-deficient mice following migration of *N. brasiliensis* larvae, was accompanied by a significant reduction in eosinophils [52]. While the failure to repair in this study could be attributed to reduced AAMΦ and ILC2s, eosinophil derived-IL-4/13 or other eosinophil derived factors may also be critical players.

Aside from type 2 cytokines, many other wound healing regulators, such as RELMα, TGFα and TGFβ, fibroblast growth factors and collagen regulating enzymes are highly secreted by eosinophils. For example, at 7 days post-wounding, eosinophils peak in number and produce TGFα [130], [131], which is known to facilitate repair of cutaneous wounds [132]. Key to the proposed mechanisms by which eosinophils may regulate wound healing, is studies that describe an interaction between eosinophils and fibroblasts promoting proliferation and matrix production [133]. Eosinophil derived products such as eosinophil cationic protein may also be key players in regulation of the extracellular matrix [134]. Interestingly, alarmin IL-33 together with IL-31 was recently suggested to activate the eosinophil–fibroblast interaction [135]. These observations together with the fact that IL-33 activates eosinophils [136] and is a key cytokine in altering the immune systems to injury as discussed above, strongly implicates eosinophils as significant players in wound repair.

Despite overwhelming circumstantial evidence that eosinophils will contribute to repair, genetic approaches to explore the role of IL-5/eosinophils in parasite killing have generally failed to see an impact of eosinophils in wound repair. In fact the converse has been shown, with IL-5-transgenic mice displaying a delay in incisional wound repair [137]. The repair-related properties of eosinophils are so diverse and likely implicate remodeling of extracellular matrix, which involves both breakdown and synthesis. Thus, their contribution to repair may be time point and context dependent – potentially promoting or slowing the repair process. It is striking that in both wound repair and parasite killing models, the data has often been contradictory. It is almost certain that this disparity is due to the multi-functionality of eosinophils and that for each model, the contribution of eosinophils will need to be dissected at each time point and with a good understanding of cellular cross-talk. What is abundantly clear is that there is an enormous opportunity in the area of eosinophil biology to gain new insight into repair and regeneration processes, especially in the context of type 2 immunity.

## 5 The effector molecules: two examples

### 5.1 Arginase

Arginase 1 provides an ideal example of a molecule with multiple functions in the context of type 2 immunity. One of the first proteins described in association with alternative macrophage activation [138], arginase 1 remains a paradigm for the cross-regulatory nature of classical vs. alternative activation. Because arginase competes with iNOS for their mutual substrate arginine, arginase suppresses the NO mediated anti-microbial pathways of classically activated macrophages. Thus suppression of inflammatory pathways is one of the key functions of arginase 1. Importantly, this occurs not only through inhibition of iNOS but by direct effects on T cell function. T cells are exquisitely sensitive to arginine concentration and depletion of arginine through arginase activation results in impaired T cell function [139], [140], [141].

Another well established property of arginase 1 is that of tissue remodeling and repair. This is because ornithine generated by arginase activity can be converted to polyamines and proline, supporting cell proliferation and collagen synthesis respectively [142]. These properties also explain the frequent association of arginase with fibrosis and in particular asthma, where it is believed to contribute to pathological tissue remodeling [68]. More unexpected, has been the finding that arginase acts as effector molecule against nematode infection. In models of secondary infection, Anthony et al. [2] demonstrated that arginase inhibition prevented worm expulsion of *H. polygyrus*, while Obato-Ninomiya et al. revealed a critical role for macrophage-derived arginase in trapping *N. brasiliensis* larvae in the skin [143]. The mechanism by which arginase enhances worm expulsion or killing is to yet established but direct effects on the worm are supported by work showing that the arginase 1 product L-ornithine inhibited the motility of *H. polygyrus* larvae [55]. Thus, the arginase metabolite L-ornithine which is a critical substrate for the wound healing machinery [96] also has anti-nematode effector function.

Arginase is one of the few examples of type 2 effectors where we have some handle on the basis for differential function. Using macrophage specific arginase deficient mice, the Wynn group demonstrated that the T cell suppressive but not the pro-repair/fibrosis functions were mediated by macrophages [144]. They postulate that fibroblasts are the critical arginase source for the promotion of collagen deposition. Further, the protective functions of arginase producing macrophages may be tissue-specific as mice lacking arginase producing macrophages had exaggerated inflammatory responses in a model of liver fibrosis [144] but unaltered inflammatory responses in several lung models [145]. Importantly, although arginase is induced by IL-4 and IL-13 and thus associated with type 2 immunity it can also be induced by microbial stimuli [146] and likely serves anti-inflammatory and repair functions in these contexts as well.

### 5.2 Amphiregulin

Another example of a single molecule performing wound repair, inflammatory suppression and anti-nematode effector function is the EGF-like growth factor, Amphiregulin (AREG). AREG is an EGF-receptor ligand expressed by a range of immune cells associated with type 2 immunity, including Th2 cells, mast cells and ILC2s [52], [147], [148]. The high level identity between mouse and human AREG and the wide clinical use of EGF-R antagonists make AREG an attractive research subject.

In 2006, Zaiss and colleagues [147] demonstrated using gene-deficient mice that AREG was needed for efficient expulsion of the gastrointestinal nematode, *Trichuris muris*. The failure to expel the parasite in AREG−/− mice was associated with a reduced proliferation of gut epithelial cells, a process known to be important for resistance to *T. muris* [149]. Subsequently several groups demonstrated an important role for AREG in tissue repair and homeostasis. In these studies AREG from ILCs promoted lung epithelial cell integrity and airway remodeling following flu infection [150]. T regulatory cell derived AREG was needed for skeletal muscle repair [151] and administration of AREG significantly reduced the mortality of mice co-infected with influenza and Listeria by protecting against lung damage [152]. The tissue protective role of AREG in the context of type 2 immunity has been supported by recent work illustrating the central role of AREG producing ILC2s in repair of nematode induced lung damage [52]. Not surprisingly, given it's contribution to wound healing, AREG is also associated with the detrimental consequences of repair, fibrosis [153]. Finally, recent data also demonstrates that mast cell derived AREG is needed for optimal T regulatory cell function [148] and thus AREG likely contributes to the to the full type 2 triad of nematode control, wound repair and local suppression of inflammation.

## 6 Adaptive immune repair

Helminth infection models can bring clarity to our understanding of wound repair by providing an explanation for the contribution of adaptive Th2 cells to wound repair pathways. The cardinal features of adaptive immunity are antigen specificity and memory and it is in the context of continual and repeated exposure to tissue damaging parasites that we might need to remember to repair our wounds in an antigen specific manner [12]. Thus Th2 cells would function to both accelerate repair and worm expulsion or killing. Although Th2 cells are an obvious source of IL-4 or IL-13 that mediates worm killing or tissue repair, other innate cells make important contributions [124] and in particular ILC2s seem to be essential in some settings [52]. However, a reliance solely on innate sources cannot explain data in which there is a failure of lungs to repair efficiently in *N. brasiliensis*-infected SCID mice and alternative activation cannot be sustained beyond the early innate response [154]. In a model of filarial nematode infection using mice lacking either RAG genes or class II, there is a striking lack of macrophages, and the few that are there do not alternatively activate [69]. Similarly, macrophages fail to proliferate in *L. sigmodontis* infected RAG deficient mice and alternative activation and IL-4 dependent proliferation occur only after the onset of the adaptive immune response [62], [94]. Additionally, macrophage proliferation requires higher doses of IL-4 than induction of RELMα, Ym1 or arginase [62] suggesting that proliferation may be particular dependent on close cellular interactions. Together the data suggest macrophage proliferation and in some contexts alternative activation requires cognate interaction that would be provided by the T cell receptor. Nonetheless, IL-4Rα activation of macrophages does occur in settings without adaptive immune response [69], [154], and in many contexts innate cells will suffice. It may be that T cells are needed for specific functions, such as macrophage proliferation and IgE production [124]. The challenge will be to define the balance of T-helper vs innate cells in different circumstances, and critically to determine whether there is an important adaptive component to tissue repair in the absence of helminth infection.

### 6.1 Antibodies

Antibodies are the quintessential adaptive immune effector molecules, and are a central feature of Th2 immunity. A role for antibody in immune protection against helminth re-infection has long been established but the need for antibody in primary infection is less consistent and likely parasite and tissue dependent. The contribution of antibody to anti-helminth immunity was reviewed recently [155]. With the close link between parasite control and tissue repair, the expectation would be that antibody would also be involved in promoting repair. Recent data from the Harris lab, demonstrates that this is indeed the case [55]. Using mice that lack antibodies (JH−/−) or activating Fc receptors (FcRγ−/−), they demonstrate in a model of secondary *H. polygyrus* infection that antibodies activate macrophages to trap and immobilize infective larvae and thus prevent parasite-induced damage. Macrophage activation via antibody led to the induction of tissue repair genes and more limited tissue damage. Of note, the ability of antibodies to induce arginase in this system was independent of the IL-4Rα, but in its absence macrophages fail to accumulate at the infection site. Thus, Th2 cytokines may be necessary for generating sufficient macrophage numbers, as well as an appropriate antibody response but induction of the key effector molecules such as arginase may occur through alternative routes.

The study described above was the first to demonstrate the dual contribution of antibody to parasite control and tissue repair. IgE, in particular, is the direct result of B-cell class switching in response to Th2 cytokines [155]. IgE is almost universally associated with helminth infection and is strongly indicative of adaptive type 2 immune activation in the host. Two recent studies on IgE, that do not involve helminths, provide new insight into the role that antibody may play in tissue protection [156], [157]. IgE is, of course, best known as the key mediator of pathological allergic reactions in atopic individuals. In these two studies, mice exposed to bee venom mounted Th2 responses and the IgE response was protective against challenge. Palm et al. demonstrated the involvement of IL-33 as a sensor of the tissue damage caused by the bee venom [156]. Thus, a major function of IgE and antibodies may be to protect against future damage associated with a particular insult such as venom or toxins. The strong association of IgE with helminth infection may be because these pathways first evolved to neutralize proteins that cause damage, such as the parasite proteases used to migrate through the tissue. IgE, however, can also directly target the parasite. Thus, antibody responses, like many other aspects of type 2 immunity such as collagen deposition, mucus production and epithelial cell turnover, may have been mechanisms of protection against tissue injury, that evolved into anti-worm effector responses to further promote host fitness.

## 7 Conclusions

As this review illustrates, Th2 associated molecules have multiple functions, with many actively contributing to both repair and parasite control. How does a single molecule perform these distinct functions? In some cases, it may be that the actual physiological mechanism is the same. For example, amphiregulin's pro-repair and pro-nematode expulsion properties may reflect a common effect on epithelial cell proliferation [147]. However, it is unlikely to be that straightforward. For example, arginase activity results in a several downstream effectors that may each have different properties and Wynn and colleagues have elegantly illustrated that arginase source is a critical determinant of function [144]. Other factors including necessary co-factors, timing, receptor availability and whether the effector molecule is soluble or membrane bound will determine the ultimate outcome of a particular effector molecule in each setting. Whether there are common rules that apply to many type 2 molecules involved in both repair and parasite control has yet to be determined.

This review has focussed on the overlapping functions of type 2 immunity in tissue repair and control of helminth parasites. However, it is now recognized that type 2 immunity and in particular AAMφ contribute to a wide range of process that include metabolic regulation, adaptive thermogenesis and tumor progression [158]. For just one example, the ability of IL-33 to promote the alternative activation of macrophages, results in outcomes that go well beyond that of wound repair or parasite control and include a protective effect on atherosclerosis [159]. It is likely, that these other functions are all tied to evolutionary adaptations of IL-4 as a tissue protective pathway. By deciphering the ancient evolutionary relationship between immunity to parasites and adaptive immune repair, we may learn valuable lessons for both the protection against helminth infection and pathways to promote healthy tissues.
